# Prenatal Detection of Cardiac Anomalies in Fetuses with Single Umbilical Artery: Diagnostic Accuracy Comparison of Maternal-Fetal-Medicine and Pediatric Cardiologist

**DOI:** 10.1155/2014/265421

**Published:** 2014-03-02

**Authors:** Ilir Tasha, Rachel Brook, Heidi Frasure, Noam Lazebnik

**Affiliations:** ^1^Spitalli Universitar Obstetrik-Gjinekologjik “Koco Gliozheni,” Bulevardi “Bajram Curri,” Tirana, Albania; ^2^Department of Obstetrics and Gynecology, University Hospitals Case Medical Center, 11100 Euclid Avenue, Cleveland, OH 44106, USA

## Abstract

*Aim*. To determine agreement of cardiac anomalies between maternal fetal medicine (MFM) physicians and pediatric cardiologists (PC) in fetuses with single umbilical artery (SUA). *Methods*. A retrospective review of all fetuses with SUA between 1999 and 2008. Subjects were studied by MFM and PC, delivered at our institution, and had confirmation of SUA and cardiac anomaly by antenatal and neonatal PC follow-up. Subjects were divided into four groups: isolated SUA, SUA and isolated cardiac anomaly, SUA and multiple anomalies without heart anomalies, and SUA and multiple malformations including cardiac anomaly. *Results*. 39,942 cases were studied between 1999 and 2008. In 376 of 39,942 cases (0.94%), SUA was diagnosed. Only 182 (48.4%) met inclusion criteria. Cardiac anomalies were found in 21% (38/182). Agreement between MFM physicians and PC in all groups combined was 94% (171/182) (95% CI [89.2, 96.8]). MFM physicians overdiagnosed cardiac anomalies in 4.4% (8/182). MFM physicians and PC failed to antenatally diagnose cardiac anomaly in the same two cases. *Conclusions*. Good agreement was noted between MFM physicians and PC in our institution. Studies performed antenatally by MFM physicians and PC are less likely to uncover the entire spectrum of cardiac abnormalities and thus neonatal follow-up is suggested.

## 1. Introduction

A normally formed umbilical cord contains two umbilical arteries and one umbilical vein. A single umbilical artery (SUA) is the most common anatomical abnormality of the umbilical cord. It is found in 0.08% to 1.90% of all pregnancies [1]. Currently, the most effective method for prenatal screening of congenital anomalies is the second trimester detailed ultrasound study. The study is performed by Maternal-Fetal-Medicine (MFM) physicians and radiologists. When properly performed, ultrasound studies will successfully reveal SUA in most pregnancies. The success rate is affected by the gestational age, maternal abdominal wall thickness, presence of a lower abdominal scar, fetal position, amniotic fluid volume, vessel tortuosity, scanning experience and skill, and lateral resolution of the equipment [2, 3]. SUAs have been associated with fetal aneuploidy, premature delivery, stillbirths, low birth-weight, and multiple congenital anomalies (including cardiac, renal, and musculoskeletal [4–6] structures). Congenital anomalies among fetuses with a SUA have been reported to be as high as 46% [6], with 31% of fetuses with a SUA having a congenital cardiac anomaly [4, 5].

The policy at University Hospitals Case Medical Center, Cleveland, Ohio, following a diagnosis of SUA in a fetus by the MFM physicians has been mandatory referral to the pediatric cardiology unit specializing in fetal and pediatric cardiac echocardiography in order to rule out any cardiac anomalies. The referral occurs regardless of whether heart or other congenital anomalies are detected. The rationale for this is that cardiac anomalies are more difficult to detect by ultrasound study due to the complexity of the human heart, especially during the gestational age of 18–20 weeks, when the great majority of pregnant women are referred for comprehensive fetal study. Pediatric cardiologists typically prefer to study the fetus at 22–24 weeks once the fetal heart and vessels are larger. Thus, within many tertiary-level pediatric hospitals, the expertise of these highly trained and skilled physicians is applied to study the fetal heart as needed.

As ultrasound technology improved and sophisticated ultrasound machines became available, many sonographers and sonologists became highly skilled in diagnosing congenital anomalies including the cardiac anomalies. More than 20 years ago Buskens et al. [7] have examined the overall diagnostic efficacy of routine fetal ultrasound screening for congenital heart disease in normal pregnancy. Since then, several studies have looked into the accuracy and agreement of *in utero* diagnosis of suspected structural cardiac anomalies diagnosed by antenatal ultrasound study performed by OBGYN/MFM physician and compared them with echocardiography performed by pediatric cardiologists antenatally or neonatally [8–12]. Diagnosis agreement in these studies ranged 38% to 100%. Some of the authors concluded that fetal echocardiography by pediatric cardiologists adds little to the care of women with no suspected heart disease on a detailed anatomic survey by OBGYN/MFM physician [9, 10], while others concluded that improved accuracy in diagnosis can be achieved through a pediatric cardiologist with special skills in fetal echocardiography working collaboratively with obstetric sonographers and sonologists to optimize the details of diagnosis [8, 9, 11, 12]. Naturally, the latter studies had lower level of diagnostic accuracy and agreement in comparison to the former studies. The studies differ in their clinical indication for fetal cardiac study, methodology, and confirmation follow-up study through neonatal echocardiography.

We were unable to identify prior studies in which patients were referred for pediatric fetal echocardiogram following diagnosis of a noncardiac anomaly (SUA), in order to compare cardiac anomaly diagnostic accuracy antenatally and confirmed with postpartum neonatal echocardiography between MFM physicians and pediatric cardiologists. In this study we took advantage of the mandatory referral policy in our institution to compare the degree of agreement between MFM physicians and pediatric cardiologists in their diagnosis of cardiac anomalies in fetuses.

## 2. Materials and Methods

The computerized archiving and reporting system of the Fetal Imaging Unit at University Hospitals Case Medical Center, Cleveland, Ohio, between January 1999 and October 2008 was searched using the words “single umbilical artery” to identify all fetuses diagnosed with a SUA. The initial MFM performed fetal ultrasound study report in which SUA was diagnosed and the pediatric echocardiography report of all cases included (antenatal and neonatal studies) were reviewed. Finally, the delivery and the newborns records were reviewed to confirm the diagnosis of SUA and the presence of or absence of a cardiac anomaly following delivery.

During the study period, the MFM physicians used ACUSON Sequoia ultrasound machine with ACUSON V5—Vector array transducer with a frequency range of 3.5/4.0/5.0 MHz and three types of General Electric ultrasound machines: Voluson 730, Voluson 730 Expert, and Voluson E8. The frequencies of the GE transducers were 4–8 MHz and 2–5 MHz. The pediatric cardiologists used various models of ACUSON Sequoia 512. The frequencies of the transducers were Acuson C7—Curved array with a frequency range of 7.0/5.0 MHz, and Acuson V5—Vector array with a frequency range of 3.5/4.0/5.0 MHz.

Data collected included race, gender, age, gestational age at diagnosis, ultrasound imaging findings, fetal echocardiogram findings, findings at delivery along with fetus survival, and neonatal echocardiogram findings. The pathology reports of the placenta and umbilical cord including the cord vessels were reviewed and entered into the database.

The second trimester ultrasound study by the MFM physicians was performed according to guidelines published by the American Institute of Ultrasound in Medicine [13]. The fetal heart study included the following: A four-chamber view of the fetal heart, visualization of the pulmonary artery and the aorta, (right and left outflow tract, resp.), the aortic and ductal arches, the 3-vessel view of the heart (pulmonary artery, aorta, and superior vena cava) documentation of the pulmonary artery bifurcation into the right and left pulmonary arteries, and the pulmonary veins, as well as normal motion of the mitral, tricuspid, pulmonary, and aortic valves. Color Doppler study was performed routinely to study the integrity of the septum between the right and the left atria and ventricles. Pulse Doppler study of the outflow tracts and valves as well as the mitral and tricuspid valves was performed if clinically indicated.

In order for a case to be included in the current study, the following criteria were used: (1) detection of a single umbilical artery by ultrasound study was performed by MFM physician in the Fetal Imaging Unit and delivery at University Hospitals Case Medical Center, Cleveland, Ohio; (2) follow-up fetal heart study was performed antenatally in the echocardiography lab at Rainbow Babies and Children Hospital Case Medical Center and neonatal confirmation of the cardiac anomaly following delivery was available; and (3) postpartum confirmation of a single umbilical artery was done by clinical inspection and histopathologic examination. The data analyzed included only live births. Cases of fetal death and termination of pregnancy due to aneuploidy or anomalous fetuses were excluded.

Subjects were divided into four groups. Group A consisted of fetuses with an isolated SUA and no additional congenital anomalies. Group B consisted of fetuses with SUA and congenital cardiac anomaly only. Group C consisted of fetuses with SUA as well as multiple congenital anomalies without cardiac anomaly. Group D consisted of fetuses with SUA and multiple congenital malformations including cardiac anomaly.

### 2.1. Statistical Analysis

Follow-up rates for presence of a pediatric cardiac study were compared between Groups A and D by use of chi-square test for frequency data. Percent agreement and 95% confidence intervals (by group and overall) for positive findings were calculated for those subjects who had both a MFM cardiac study and a pediatric cardiac study performed. The kappa statistic was calculated to describe interrater reliability and account for agreement beyond chance [14]. The kappa statistic can be interpreted as values < 0 as indicating no agreement and 0–0.20 as slight agreement, 0.21–0.40 as fair agreement, 0.41–0.60 as moderate agreement, 0.61–0.80 as substantial agreement, and 0.81–1 as almost perfect agreement. Sensitivity, specificity, and positive and negative predictive values with 95% confidence intervals were calculated by comparing the results from the MFM performed ultrasound study with those performed by the pediatric echocardiography. For this calculation, the pediatric echocardiography study was used as the “gold standard” comparison.

The study was approved by the Institutional Review Board (IRB) of University Hospitals Case Medical Center, Cleveland, Ohio. The IRB waived HIPAA authorization or consent required for access to and use of patients records since this study was a retrospective review.

## 3. Results

A total of 39,942 pregnant women were studied between January 1999 and October 2008 by MFM physicians in the Fetal Imaging Unit at University Hospitals Case Medical Center, Cleveland, Ohio. In 376 (0.94%) cases, the ultrasound study noted SUA. Of those 376 fetuses, 182 (48.4%) met the inclusion criteria (Table 1). The contributing reasons for lack of pediatric echocardiography follow-up among patients diagnosed by MFM physicians to have cardiac anomalies were: Complex congenital anomalies resulting in elective termination of pregnancy, spontaneous intrauterine death, patient choice to not pursue follow-up fetal echography and delivery outside our institution.

The follow-up rate of fetal echocardiogram in the Pediatric Cardiology Unit varied between groups (Table 1; *P* = 0.014). Specifically there was a difference between the follow-up rate in group B as compared to Groups A, C, and D. The highest (91.6%) follow-up rate was seen amongst subjects diagnosed with SUA and isolated cardiac anomaly (Group B). There was no difference between the follow-up rates observed between Groups A, C, and D.

The182 fetuses that met all inclusion criteria were divided into four groups as specified in Table 2. 

*Group A.* Of the 114 fetuses in this group 112 had unremarkable pediatric fetal echocardiogram. Thus, 98.2% agreement between the two cardiac studies is noted for this group. 

*Group B.* Of the 11 fetuses in this group 10 cases were also shown to have cardiac anomaly by the pediatric fetal echocardiogram for an agreement of 90.8% (10/11). One fetus diagnosed with a VSD by the MFM physicians was found to have normal fetal heart by the pediatric cardiology team and was confirmed to have unremarkable cardiac study in early neonatal life. In 7 of the 10 fetuses with abnormal cardiac examination by MFM physicians, additional cardiovascular abnormalities were detected on the follow-up pediatric echocardiogram. Of note is the fact that 5 of these 7 fetuses were found to have the additional cardiac malformation only on the postdelivery echocardiography. 

*Group C.* All 24 fetuses with SUA and multiple congenital anomalies but no cardiac anomaly on the study performed by the MFM physicians had unremarkable pediatric fetal echocardiogram studies for agreement of 100%. There were two cases in Group C where both the MFM physician and the pediatric cardiologist failed to detect significant cardiac malformation in the same 2 cases (2/182 = 1.1%). The cardiac abnormalities were later correctly diagnosed by the pediatric cardiologist following birth. These two cases consisted of coarctation of the aorta in one fetus and Tetralogy of Fallot with mild pulmonary artery stenosis and VSD in the other. Both cases were extremely challenging due to poor maternal acoustic properties. 

*Group D*. Thirty-three fetuses were noted to have SUA as well as abnormal cardiac findings and additional noncardiac abnormal findings when studied by the MFM physicians. Only 25 fetuses were confirmed by pediatric fetal echocardiogram to have cardiac abnormality for agreement of 75.8% (25/33). Of the 25 fetuses the pediatric echocardiogram noted additional cardiovascular abnormalities in 36% (9/25). In 7 of the 9 fetuses, the additional abnormal findings were appreciated only on postdelivery neonatal echocardiography.

The specific cardiac abnormalities found, including the additional cardiac anomalies noted by the pediatric cardiologists, are listed in Tables 3 and 4.

Agreement between MFM physicians and pediatric cardiologists in all groups combined was 94% (171/182) (95% CI [89.2, 96.8]) as seen in Table 5. The computed kappa statistic was 0.825; 95% CI (0.726, 0.925), considered to be “very good agreement beyond chance,” when compared to common interpretation guidelines for the kappa statistics.

Data for all 182 fetuses regarding the presence or absence of cardiac abnormality as determined by the MFM physicians versus the “gold standard” fetal and neonatal echocardiogram performed by pediatric cardiologists are listed in Table 5. The overall incidence of confirmed cardiac abnormalities among fetuses with SUA was 20.3% (37/182). When comparing MFM physicians performing fetal ultrasound study versus fetal echocardiogram performed by the pediatric cardiologists (considered the gold standard for this study) in our institution the sensitivity was 35/38 (92.1%; 95% CI [77.5, 97.9]), and specificity of 136/144 (94.4%; 11 95% CI [89.0, 97.4]. The positive and negative predictive values were 35/43 (81.4%; 95% CI [66.1, 91.1]) and 136/139 (97.8%; 95% CI [93.3, 99.4], respectively. The MFM physicians overdiagnosed cardiac anomalies in 8/182 (4.4%) cases, a single case in Group B and 7 cases in Group D. In two cases, both groups of physicians, MFM and pediatric cardiologists, failed to diagnose antenatally significant cardiovascular abnormalities in the same 2 fetuses, coarctation of the aorta in one and Tetralogy of Fallot in the other. These 2 cases were not included in the calculations comparing the two groups of physicians but were included in calculating the total number of cardiac anomalies among fetuses with SUA in this study.

## 4. Discussion

In this study, we took advantage of the mandatory referral policy for pediatric cardiac echocardiography following diagnosis of SUA in a fetus by the MFM physicians to compare the degree of agreement between MFM physicians and pediatric cardiologists. Since the study period spans over 9 years, it involves multiple physicians performing ultrasound studies. Thus, the comparison between the two imaging services is the summation of expertise of multiple participating sonographers and physicians.

The incidence of cardiac abnormalities among fetuses with SUA was 20.3% (37/182), well in accord with previously published studies that reported incidence ranging from 1% to 32% [4–6].

Our data suggests that in our institution once an isolated single umbilical artery (i.e., no other abnormality) has been diagnosed by MFM physician, the risk of completely underdiagnosing a significant congenital cardiac anomaly is low 1.1% (2/182). Of the 35 fetuses with confirmed congenital cardiac abnormalities by pediatric cardiologists (gold standard) 33 (94.2%) fetuses were first diagnosed by the MFM physicians, similar to that reported by Gossett et al. study of 1/18 (95%) [15]. The 2 cases of missed cardiac abnormalities on studies performed by the MFM physicians were among fetuses in Group A. They were each found to have muscular ventricular septal defect on the pediatric fetal echocardiogram.

In 8 fetuses, the MFM physicians diagnosed a form of congenital cardiac abnormality which could not be confirmed by the pediatric cardiologists. The “false positive” diagnosis by the MFM physician of a ventricular septal defect might be explained by the fact that in 74% of pregnancies in which an isolated fetal ventricular septal defect was diagnosed the defect resolved spontaneously before birth [16, 17]. In addition, bias among the MFM physicians to overdiagnose cardiac anomalies when SUA combined with multiple other congenital defects were found should also be considered. Review of the cases misdiagnosed with ventricular septal defect noted incorrect angle of insulation and level of heart and vessels study as well as incorrect placement of the Doppler gate might explain the false reading of the recorded waveform.

An important fact uncovered by this study is that pediatric echocardiogram is more likely to diagnose additional cardiac and vascular abnormalities beyond those already identified by the MFM physicians. Most of the additional abnormal findings were not detected by the pediatric cardiologists in the antenatal fetal echocardiogram but rather only in the postdelivery neonatal echocardiography. The overall higher rate of diagnosis by the pediatric cardiologists might be explained by the fact that, in most cases, antenatal pediatric fetal echocardiogram was performed at about 22 weeks or later while the initial MFM study was performed at 16–21 weeks for the great majority of cases. One would expect that the optimal technical study terms in the early neonatal period would result in better diagnosis when performed by experienced and skilled pediatric cardiologist as has been previously reported [8, 9, 11, 12]. This is further supported by the two cases in which both the MFM physicians and the pediatric cardiologists failed to correctly identify major cardiac abnormalities in the same two fetuses. These cases were later diagnosed on the post-delivery follow-up pediatric echocardiogram. It should be noted that in, both cases, the maternal body habitus was a major negative factor limiting the quality of the study as both women were insulin dependent diabetics with BMI > 48.

An important finding of our study is the difference in patients' compliance with the recommendation for antenatal pediatric fetal echocardiogram follow-up. Compliance varied significantly among the different 4 groups and was mainly related to whether a cardiac defect or any other congenital anomalies were detected on the initial ultrasound imaging performed by the MFM physicians. It might reflect subject bias in accepting and complying with the recommendation for a follow-up visit with the pediatric cardiology service in view of their understanding of the severity of the abnormalities found. This was more pronounced in Group D, which consisted of fetuses with cardiac anomaly in addition to multiple other congenital malformations. This in turn could have introduced certain bias in the nature and or severity of cases followed up by the pediatric cardiologists.

Recently Trivedi et al. [12] published a study aiming to determine the variation between prenatal diagnosis (by sonography) and postnatal diagnosis (neonatal echocardiography, cardiac catheterization, or autopsy) of congenital cardiac lesions diagnosed by both, MFM physicians and pediatric cardiologists. Unlike our study where patients with a noncardiac anomaly (SUA) were first studied by MFM physicians and then by the pediatric cardiologists, Trivedi et al. included only subjects with already diagnosed or suspected fetal cardiac abnormality or subjects with documented cardiac anomaly by autopsy or neonatal follow-up. In spite of this major deference, some similarities do exist between our findings and those of Trivedi et al. In the study by Trivedi et al., the detection rates of congenital heart disease were not statistically different between MFM physicians and pediatric cardiologists, 77.9% (46 of 59) versus 85.0% (34 of 40 *P* = 0.3) similar to our study as noted by the overall agreement for all groups (Table 5).

There are several limitations to our study. Despite a larger sample relative to most previously published retrospective studies [8–12, 15], our population size is still relatively small and thus the incidence of both underdiagnosed fetal cardiac abnormalities and the expected percentage of cardiac anomalies among fetuses with isolated SUA cannot be correctly established. As this is a retrospective, unblinded study, the fact that SUA has been diagnosed in a given fetus introduces the risk of bias for the MFM physician performing the study, as the physician is primed to “look harder” for cardiac anomaly, perhaps even more so for the pediatric cardiologist who is aware of the MFM physician's prior diagnosis. In addition, the gestational age at which the study was performed by the two imaging services varies in accordance with patients' compliance with prenatal care. However, this problem is inherent to any retrospective study.

While some of our study findings are similar to findings reported by other researchers [6–10, 12, 15], one should be cognizant of the fact that those studies were conducted in tertiary-care institutions with highly experienced MFM and pediatric cardiology physicians. Therefore, the decision to consider mandatory follow-up by pediatric fetal echocardiogram should depend on similar studies that should be conducted in each institution. The timing of pediatric echocardiogram to be performed, prenatally or after delivery, remains unresolved and requires future studies. Until such studies will be available, it appears prudent to consider follow-up echocardiography in the very early neonatal period for all newborns suspected of having congenital cardiac anomaly and in cases where optimal cardiac views were unachievable.

In summary, our study reveals good agreement between MFM and pediatric cardiology physicians in detecting fetal cardiac anomalies in fetuses with SUA. Studies performed antenatally by MFM physicians and pediatric cardiologists are less likely to uncover the entire spectrum of cardiac abnormalities within each affected fetus. In addition, overdiagnosis of congenital cardiac anomaly by MFM physician is noted among fetuses with multiple other anomalies. Thus, follow-up study with pediatric cardiology may be warranted for all fetuses with SUA or when cardiac anomaly is suspected. The decision to consider mandatory follow-up by pediatric fetal echocardiogram should depend on proven expertise in each institution.

## Figures and Tables

**Table 1:** Follow-up rates once MFM performed fetal ultrasound study have diagnosed SUA and fetal echocardiogram performed by pediatric cardiologist.

Group	MFM antenatal study	Follow-up pediatric fetal cardiac study	Follow-up rate (%)	95% confidence interval
A	253	114	45.0	38.9, 51.4
B	12	11	91.7	59.8, 99.6
C	45	24	53.3	38.0, 68.1
D	66	33	50.0	37.5, 62.4
Total	**376**	**182**	**48.4**	**43.3, 53.6**

Chi-square = 10.6; *P* = 0.014; Group A: fetuses with isolated SUA; Group B: fetuses with SUA and congenital cardiac anomaly only; Group C: fetuses with SUA and multiple congenital anomalies without cardiac anomaly; Group D: fetuses with SUA and multiple congenital malformations including cardiac anomaly.

**Table 2:** Comparison of fetuses with cardiac anomaly found by MFM physician versus pediatric cardiologist for Groups A through D (*n* = 182).

	Positive cardiac study FindingsMFM physicians	Positive cardiac study FindingsPediatric cardiologists	Agreement %	95% Confidence interval
Group A *N* = 114	0	2	98.2% (112/114)	93.2, 99.7
Group B *N* = 11	11	10	90.8% (10/11)	57.2, 99.5
Group C *N* = 24	0	0 (+2*)	100% (24/24)	82.3, 100
Group D *N* = 33	33	25	75.8% (25/33)	57.4, 88.3
Overall	**44**	**35** (+2*)	**94.0% (171/182)**	**89.2, 96.8**

*Note: 2 cases of cardiac anomalies were missed by both imaging services and were only diagnosed following delivery. Group A: fetuses with isolated SUA; Group B: fetuses with SUA and congenital cardiac anomaly only; Group C: fetuses with SUA and multiple congenital anomalies without cardiac anomaly; Group D: fetuses with SUA and multiple congenital malformations including cardiac anomaly.

**Table 3:** Cardiac abnormalities detected by MFM physicians and pediatric cardiologists for fetuses in Group B.

Case	MFM performed comprehensive fetal ultrasound study	Cardiac diagnoses via pediatric fetal echocardiogram	Additional abnormal cardiac findings on pediatric fetal and neonatal echocardiogram
1	Hypoplastic left heart, mitral stenosis, tricuspid insufficiency, pericardial effusion, and bradycardia	Hypoplastic left heart, mitral stenosis, tricuspid insufficiency, pericardial effusion, and bradycardia	Dilated coronary sinus with left superior vena cava returning to coronary sinus
2	Double-outlet right ventricle w/VSD	Double-outlet right ventricle w/VSD	Interrupted aortic arch
3	VSD w/pericardial effusion	VSD w/pericardial effusion	
4	Hypoplastic right ventricle and pulmonary artery, single pulmonary vein, and a VSD	Hypoplastic right ventricle and pulmonary artery, single pulmonary vein, and a VSD	ASD with atrial septal aneurysm
5	VSD	VSD	Coarctation of the aorta
6	Hypoplastic left heart	Hypoplastic left heart	Restrictive foramen ovale
7	Hypoplastic left ventricle, aortic stenosis/atresia, mitral stenosis/atresia, pulmonary stenosis/atresia, abnormal location of ductus venosus	Hypoplastic left ventricle, aortic stenosis/atresia, mitral stenosis/atresia, pulmonary stenosis/atresia, abnormal location of ductus venosus	Fibroelastosis
8	Small VSD	Unremarkable study	Unremarkable study
9	Hypoplastic left heart	Hypoplastic left heart	
10	Tetralogy of Fallot	Tetralogy of Fallot	
11	Hypoplastic right heart	Hypoplastic right heart	Subaortic stenosis

**Table 4:** Cardiac abnormalities detected by MFM physician versus pediatric cardiologist performed fetal echocardiogram for Group D.

Case	MFM performed comprehensive fetal ultrasound study	Cardiac diagnoses via pediatric fetal echocardiogram	Additional abnormal cardiac findings on pediatric fetal and neonatal echocardiogram
1	VSD, double-outlet right ventricle	VSD, double-outlet right ventricle	Subaortic stenosis
2	VSD with echogenic intracardiac focus	Echogenic intracardiac focus only	
3	Tetralogy of Fallot with pulmonary artery hypoplasia	Tetralogy of Fallot with pulmonary artery hypoplasia	
4	VSD, pentalogy of Cantrell, absent ductus venosus	VSD, pentalogy of Cantrell, absent ductus venosus	Complete common atrio-ventricular canal defect
5	VSD	VSD	Mild left heart hypoplasia
6	VSD, hypoplastic aortic arch, and aortic stenosis	VSD, hypoplastic aortic arch, and aortic stenosis	
7	Mildly hypoplastic right ventricle w/small pericardial effusion	Unremarkable study	
8	Partial atrioventricular septal defect	Partial atrioventricular septal defect	Mitral valve regurgitation
9	Mildly hypoplastic left ventricle w/small VSD	Unremarkable study	
10	Hypoplastic left atrium w/mitral stenosis or atresia and VSD, suspected segmental stenosis in the inferior vena cava	Hypoplastic left atrium w/mitral stenosis, VSD	Absent renal to hepatic inferior vena cava segment with azygous continuation
11	Hypoplastic left heart with VSD	Hypoplastic left heart w/VSD	
12	VSD narrow and elongated LVOT	VSD and elongated LVOT	Atrioventricular septal defect
13	Mild pulmonic insufficiency w/mild pulmonary artery dilation	Unremarkable study	
14	Hypoplastic left ventricle	Hypoplastic left ventricle	Small VSD and coarctation of the aorta
15	Overriding aorta with VSD	Overriding aorta with VSD	
16	Cardiomegaly	Unremarkable study	
17	Unremarkable study	Biventricular hypertrophy, small pericardial effusion	
18	Atrioventricular canal defect	Atrio-ventricular canal defect	
19	Right sided cardiomegaly	Unremarkable study	
20	Endocardial cushion defect, aortic arch hypoplasia, and aortic stenosis	Endocardial cushion defect, aortic arch hypoplasia, and aortic stenosis	
21	Hypoplastic left heart	Hypoplastic left heart	
22	Cardiac fibroelastosis with severe biventricular hypertrophy and bradycardia	Cardiac fibroelastosis w/severe biventricular hypertrophy and bradycardia	
23	Atrioventricular septal defect	Unremarkable study	
24	Hypoplastic left ventricle with double right ventricle outlet	Hypoplastic left ventricle with double right outlet ventricle outlet	
25	Biventricular hypertrophy	Biventricular hypertrophy	
26	Mild left ventricular hypoplasia	Mild left ventricular hypoplasia	
27	VSD, pulmonary stenosis/atresia	VSD, pulmonary stenosis/atresia	
28	Dextroversion of the heart w/situs solitus	Dextroversion of the heart w/situs solitus	
29	Unbalanced AV canal, hypoplastic left heart with outflow tract anomalies (dilated pulmonary artery, nonvisualized aortic tract, truncus not excluded)	Unbalanced AV canal with abnormal outflow tract versus hypoplastic left heart with outflow tract anomalies (dilated pulmonary artery, nonvisualized aortic tract, truncus not excluded), aortic stenosis, hypoplastic ascending aortic arch	
30	Narrow aortic outflow	Unremarkable study	
31	VSD	VSD	Mild right atrium dilation
32	Pericardial effusion	Pericardial effusion	
33	Single right ventricle with dextrocardia	Single right ventricle with dextrocardia	

**Table 5:** Presence (+) or absence (−) of cardiac abnormality by test: fetal echocardiogram performed by MFM versus pediatric cardiologists.

	(+) Fetal echocardiogram	(−) Fetal echocardiogram	
(+) US imaging study	35	8	43
(−) US imaging study	3	136*	139
Total number	**38**	**144**	**182**

Overall agreement between MFM and pediatric cardiology for all groups 171/182 (94.0%; 95% CI [89.2, 96.8]).

*Note: 2 cases of cardiac anomalies were missed by MFM imaging and pediatric fetal echocardiogram studies but were diagnosed following delivery.
